# Medical Degree Disparity Among Authors of Original Research in Pediatric Journals

**DOI:** 10.7759/cureus.5119

**Published:** 2019-07-10

**Authors:** Brian Merritt, Christopher F Dion, Robert Sprague, John Ashurst

**Affiliations:** 1 Osteopathic Medicine, Lake Erie College of Osteopathic Medicine, Erie, USA; 2 Emergency Medicine, McLaren Macomb Hospital, Mt. Clemens, USA; 3 Emergency Medicine, Kingman Regional Medical Center, Kingman, USA

**Keywords:** osteopathic, pediatrics, medical education, research

## Abstract

Introduction: Research and scholarly output are integral parts of residency training for both residents and faculty. With the transition to a single accreditation system, scholarly activity and output of osteopathic physicians have garnered significant interest. Previous research has shown that osteopathic physicians in emergency medicine and obstetrics and gynecology infrequently publish original research in high impact journals.

Objective: To determine whether there is a disparity between osteopathic and allopathic physicians among authors who publish original research manuscripts in three high-impact pediatric journals.

Methods: The medical degree designation of the first and senior author (last author) and any advanced degree either author may have obtained were retrieved from the Journal of Pediatrics (J Pediatr), Pediatrics, and JAMA Pediatrics (JAMA Pediatr) for the years 2000, 2005, 2010 and 2015. Data was analyzed using simple descriptive statistics and linear regression.

Results: In total, 2232 manuscripts and 4296 authors were reviewed with 0.58% (25/4296) of all authors being osteopathic physicians. A total of 0.81% (18/2232) of first authors and 0.34% (7/2064) of senior authors were osteopathic physicians. For those with a dual degree, a total of 0.64% (5/777) of first and 0.33% (3/904) of senior authors were osteopathic physicians. No statistical trend could be established for increased first (p=0.24), senior (p=0.16), dual degree first (p=0.08) or dual degree senior (p=0.06) osteopathic physician authorship. Likewise, no statistical trend for increased authorship could be established for any Doctor of Osteopathic Medicine (DO) authorship role in the three journals over the time period studied.

Conclusion: Very few osteopathic physicians have served as either the first or senior author in published original research manuscripts for the Journal of Pediatrics, Pediatrics, or JAMA Pediatrics for the years studied. Also, no statistical trend could be established for increased osteopathic physician publication over the same years.

## Introduction

Scholarly activity has been an integral component of graduate medical education since its early inception and has been a driving force in academic medicine. In 2001, the American Academy of Pediatrics (AAP) noted that only 10% of graduates from a pediatric residency pursue traditional research careers despite encouraging early research training and the establishment of defined curriculums [1]. For pediatric residencies, the Accreditation Council for Graduate Medical Education (ACGME) currently recommends that “some members of the faculty should demonstrate scholarship by one or more of the following: publication of original research or review articles in peer-reviewed journals, or chapters or textbooks…”[2]. In the last iteration of basic standards for community-based residency training in pediatrics, neither the American Osteopathic Association (AOA) nor the American College of Osteopathic Pediatricians included a recommendation of scholarly activity for faculty [3]. Instead, the organizations recommended that each resident must participate in scholarly activity as determined by the program director [3].

As the transition into a single graduate medical accreditation system occurs, research has shown that the publication of manuscripts by Doctor of Osteopathic Medicine (DO) is lacking in certain specialties as compared to their allopathic Doctor of Medicine (MD) counterparts despite ACGME recommendations [4-5]. However, no data has currently been published in the specialty of pediatrics. In this study, the authors sought to determine if there was a medical degree disparity among physician-scientists who published manuscripts in three high impact pediatric journals in 2000, 2005, 2010, and 2015.

## Materials and methods

Study design

This was a retrospective analysis of medical degree (MD vs. DO) trends in publications from the Journal of Pediatrics (J Pediatr), Pediatrics (Pediatrics) and JAMA Pediatrics (JAMA Pediatr). Journals were chosen based upon having a minimum impact factor of 3.5 (range, 3.89-9.52) and a five-year impact factor of 4.0 (range, 4.12-9.57) according to the 2015 Journal Citation Reports, as well as citation half-life of at least 15 years according to each journal’s website. The senior author trained the other authors in proper abstracting technique in person by identifying the authors and degrees for the first month of Pediatrics together. 

Only original research manuscripts from 2000, 2005, 2010 and 2015 were included in the final analysis. Research articles were reviewed to ascertain the medical degree of the first and senior author. This was determined by inspection of the abstract or manuscript that was publically available through each journal’s website. Manuscripts that were not authored by a physician were excluded from final analysis. For those with more than one advanced degree, both the medical degree and advanced degree (MPH, MSc, Ph.D., etc.) were obtained for analysis. Following final data collection, 100 total data points were chosen by a random number generator to determine inter-rater reliability. 

Data analysis

The proportions of MD and DO authorship across the years were compared by using simple descriptive statistics. The proportions of those holding both a medical degree and a second advanced degree were also analyzed using descriptive statistics. Trends in authorship were analyzed using simple linear regression. Inter-rater reliability was determined using the kappa coefficient. Statistical significance was defined as a P≤0.05.

## Results

A total of 2232 manuscripts and 4296 authors were reviewed for the years included in analysis. A total of 2232 authors were considered first authors and of these 777 authors held a dual degree. A total of 2064 authors were considered the senior author on a manuscript with 904 of these authors holding a dual degree. Inter-rater reliability for first and senior author degree designation was K=1. 

Overall, 0.58% of all authors (25/4296) were osteopathic physicians, while 99.42% (4271/4296) were allopathic physicians (Table 1). When only first authors were considered, a total of 0.81% (18/2232) were osteopathic physicians and 99.19% (2214/2232) were allopathic physicians. For those authors who held a dual degree and were the first author of a manuscript, only 0.64% (5/777) were osteopathic physicians. When the last author was considered, 0.34% (7/2064) of physicians held a degree in osteopathic medicine and the remainder were allopathic physicians. Similarly, 0.33% (3/904) of those senior authors holding a dual degree were also osteopathic physicians. When assessing each journal, the majority of first and senior authors were allopathic physicians for each year studied (Table 2). Likewise, the allopathic physicians made up the majority of physicians who held a dual degree and acted as either the first or senior author for original research manuscripts (Table 3). 

**Table 1:** Proportions of DOs and MDs Serving as First or Senior Author During All Three Years Studied J of Pediatrics: Journal of Pediatrics; DO: Doctor of Osteopathic Medicine; JAMA Pediatr: JAMA Pediatrics; MD: Doctor of Medicine.

Journal	DO or MD as First Author, % (No./All First Authors)		DO or MD as Senior Author, % (No./All Senior Authors)	
	DO	MD	DO	MD
Overall	0.81(18/2232)	99.19 (2214/2232)	0.34(7/2064)	99.66 (2057/2064)
J of Pediatrics	0.90 (7/774)	99.10 (767/774)	0.13 (1/750)	99.87 (749/750)
Pediatrics	0.70 (8/1142)	99.30 (1134/1142)	0.48 (5/1033)	99.52 (1028/1033)
JAMA Pediatr	0.95 (3/316)	99.05 (313/316)	0.36 (1/281)	99.64 (280/281)

**Table 2:** Proportions of DOs and MDs Serving as First or Senior Author by Journal and Year J of Pediatrics: Journal of Pediatrics; DO: Doctor of Osteopathic Medicine; JAMA Pediatr: JAMA Pediatrics; MD: Doctor of Medicine.

Authorship by Journal	DO or MD as First or Senior Author, % (No./All First or Senior Authors)				P Value
	2000	2005	2010	2015	
Overall					
DO authors					
First	0.57 (3/529)	0.70 (4/574)	0.49 (3/608)	1.54 (8/520)	0.24
Senior	0.21 (1/477)	0 (0/534)	0.35 (2/569)	0.83 (4/484)	0.16
MD authors					
First	99. 43 (526/529)	99.30 (570/574)	99.51 (605/608)	98.46 (512/520)	0.97
Senior	99.79 (476/477)	100 (534/534)	99.65 (567/569)	99.17 (480/484)	0.87
J of Pediatrics					
DO authors					
First	0 (0/137)	1.36 (2/147)	1.27 (3/237)	0.79 (2/253)	0.28
Senior	0 (0/150)	0 (0/138)	0 (0/213)	0.40 (1/249)	0.23
MD authors					
First	100 (137/137)	98.64 (145/147)	98.73 (234/237)	99.21 (251/253)	0.06
Senior	100 (150/150)	100 (138/138)	100 (213/213)	99.60 (248/249)	0.09
Pediatrics					
DO authors					
First	1.05 (3/285)	0.29 (1/341)	0 (0/308)	1.92 (4/208)	0.86
Senior	0.42 (1/236)	0 (0/311)	0.66 (2/301)	1.08 (2/185)	0.33
MD authors					
First	98.95 (282/285)	99.71(340/341)	100 (308/308)	98.08 (204/208)	0.41
Senior	99.58 (235/236)	100 (311/311)	99.34 (299/301)	98.92 (183/185)	0.64
JAMA Pediatr					
DO authors					
First	0 (0/107)	1.16 (1/86)	0 (0/63)	3.34 (2/59)	0.33
Senior	0 (0/91)	0 (0/85)	0 (0/55)	2 (1/50)	0.23
MD authors					
First	100(107/107)	98.84 (85/86)	100 (63/63)	96.61 (57/59)	0.02
Senior	100 (0/91)	100 (85/85)	100 (55/55)	98 (49/50)	0.57

**Table 3:** Proportions of DOs and MDs With Dual Degrees Serving as First or Senior Author by Journal and Year J of Pediatrics: Journal of Pediatrics; DO: Doctor of Osteopathic Medicine; JAMA Pediatr: JAMA Pediatrics; MD: Doctor of Medicine.

Authorship by Journal	DO or MD With Dual Degrees as First or Senior Author, % (No./All First or Senior Authors With Dual Degrees)				P Value
	2000	2005	2010	2015	
Overall					
DO authors					
First	0 (0/132)	0.57 (1/176)	0.41 (1/243)	1.33 (3/226)	0.08
Senior	0 (0/153)	0 (0/215)	0.37 (1/270)	0.75 (2/266)	0.06
MD authors					
First	100 (132/132)	99.43 (175/176)	99.59 (242/243)	98.67 (223/226)	0.11
Senior	100 (152/153)	100 (215/215)	99.63 (269/270)	99.23 (264/266)	0.07
J of Pediatrics					
DO authors					
First	0 (0/25)	0 (0/39)	1.47 (1/68)	0 (0/90)	0.74
Senior	0 (0/45)	0 (0/51)	0 (0/81)	0.81 (1/124)	0.23
MD authors					
First	100 (25/25)	100 (39/39)	98.53 (67/68)	100 (90/90)	0.01
Senior	100 (45/45)	100 (51/51)	100 (81/81)	99.19 (123/124)	0.04
Pediatrics					
DO authors					
First	0 (0/71)	0.96 (1/104)	0 (0/140)	1.85 (2/108)	0.33
Senior	0 (0/72)	0 (0/131)	0.61 (1/163)	0.90 (1/111)	0.11
MD authors					
First	100 (71/71)	99.04 (103/104)	100 (140/140)	98.15 (106/108)	0.35
Senior	100 (72/72)	100 (131/131)	99.39 (162/163)	99.10 (110/111)	0.50
JAMA Pediatr					
DO authors					
First	0 (0/36)	0 (0/33)	0 (0/35)	3.57 (1/28)	0.23
Senior	0 (0/36)	0 (0/33)	0 (0/26)	0 (0/31)	NA
MD authors					
First	100 (36/36)	100 (33/33)	100 (35/35)	96.43 (27/28)	0.20
Senior	100 (36/36)	100 (33/33)	100 (26/26)	100 (31/31)	0.32

No statistically significant trend could be established for increased DO authorship in either the first (p=0.24) or senior author roles (p=0.16) for published manuscripts over the time period studied (Table 2). Also, no trend could be established for those DOs who held a dual degree and served as either the first (0.08) or senior author (0.06) (Table 3).

## Discussion

Much like in emergency medicine and obstetrics and gynecology, osteopathic physicians rarely published original research manuscripts in the studied three high-impact pediatric journals over the time period studied [4-5]. Data has shown that of the 57,542 active physicians in pediatrics in 2015, 71.06% held an MD degree [6]. Despite the large number of osteopathic physicians in the pediatric profession, only a small amount serve as either the first or senior author on published original research manuscripts. It still remains unclear the exact reason for the degree disparity in publication but could be related to the lack of dual degree trained osteopathic physician-scientists in the working pediatric population. 

Based upon the data, very few of those DOs who published research manuscripts held a dual degree. Similar data has also been seen by those DOs who have published manuscripts in emergency medicine and obstetrics and gynecology [4-5]. Current literature shows that those pediatric hospitalists who hold a dual degree are more likely to publish original research and present research abstracts at scientific conferences [7]. Research has also shown that those holding a dual degree on average produce four manuscripts during training [7]. Without this vital scientific training and mentoring, it could be difficult osteopathic physician-scientist to publish a large volume of research [8].

Although a large number of students enrolled in osteopathic medical school have a research background, it has been hypothesized that a lack of research training during medical school may play a key role in the decreased publication rate of osteopathic physicians [9-10]. The Fresno test was designed to evaluate a learner’s knowledge in evidence-based medicine and has been externally validated [11]. Recently, it has been shown that third and fourth-year osteopathic medical students score significantly lower than their allopathic counterparts on this exam [12]. The largest difference found between the two groups was that in health numeracy including number needed to treat, confidence intervals and 2 x 2 grids [13]. Further complicating matters is that a large percentage of pediatric residents reported little interest in conducting research during residency and had only fair or poor knowledge of grant writing, statistical analysis, manuscript writing and research design [13]. Without the groundwork or drive in academic medicine, one could theorize that very few clinicians will go onto publish manuscripts.

Mentoring has been described as central to academic medicine and those with an academic mentor have been shown to have an increase in both research productivity and grant success [14]. The committee on pediatric research has also stated that “junior faculty research success is dependent on outstanding mentorship from senior faculty” [15]. Based upon our results, however, very few senior authors are osteopathic physicians. Furthermore, very few osteopathic physicians serve as the editor in chief, on the editorial boards of major academic journals or have served on a National Advisory Committee for the National Institutes of Health [16-17]. Without appropriate mentorship, many research projects may never pass the developmental phase and would not reach publication status. 

To combat the issue of low academic output, both residencies and national organizations have been conducting research in order to determine the best pathway for increased research involvement and productivity. On a national level, the Committee on Pediatric Research recommended a bottom-up approach in which scholarly activity training should begin in medical school and should be followed all the way through junior faculty status [15]. From a residency standpoint, a dedicated academic rotation has led to productive research accomplishments including presentations at conferences and publications of manuscripts [18]. Also, dedicated research tracks and a research curriculum have also shown an increase in productivity [19].

Limitations

Although a large number of data points were obtained, the authors are unable to comment on the years, or other journals that were not reviewed. The authors also cannot comment on those articles that were submitted to these journals and not accepted for final publication. Due to the nature of the study, authors who published multiple times or in multiple roles were not removed from the final analysis. This may have altered data in that one prolific author may have skewed trends in one direction. Due to the overall number of data points collected, however, it is felt this is highly unlikely. The authors also relied on each journal to publish the author's degree designation. The authors did not review each authors degree designation through an internet search but feel that inaccuracies are not likely. 

All attempts were made to include only original research manuscripts by using journal specific sections for “Original Research” but studies that were not reviewed found in other sections of the journal may have also fit into this category. Given the large number of research studies included in the final analysis, it is felt that accurate trends were established based upon available data. Other types of journal articles including case reports, clinical images, reviews and letters to the editor were not reviewed. If reviewed, a trend may have been established for osteopathic or allopathic publication over time.

## Conclusions

Osteopathic physicians, compared to MDs rarely served as either the first or senior authors in manuscripts published in three high impact pediatric journals over the years studied. No statistical trend over time could be established for publication of research articles with DO first or senior authors, including those with a dual degree.

## References

[1] Chesney R, Dungy C, Gillman M (2001). Promoting education, mentorship and support for pediatric research. Pediatrics.

[2] (2019). ACGME program requirements for graduate medical education in pediatrics. Retrieved August 15.

[3] (2019). Basic standards for community based residency training in pediatrics. Retrieved August 15.

[4] Lammers R, Simunich T, Ashurst J (2016). Authorship trends of emergency medicine publications over the last two decades. West J Emerg Med.

[5] Merritt B, Simunich T, Ashurst J (2019). Medical degree disparity among authors in obstetrics and gynecology journals. J Am Osteopath Assoc.

[6] Association of the American Medical Colleges 2016 Physician Specialty Data Report. (2019). Association of the American Medical Colleges 2016 Physician Specialty Data Report. https://www.aamc.org/data/workforce/reports/458498/1-5-chart.html.

[7] Teufel RJ, Bekmezian A, Wilson K (2012). Pediatric hospitalist research productivity: predictors of success at presenting abstracts and publishing peer-reviewed manuscripts among pediatric hospitalists. Hosp Pediatr.

[8] Bau JT, Frolkis AD, Nathoo N, Yipp BG, Hollenberg MD, Beck PL (2017). Career and research outcomes of the physician-scientist training program at the University of Calgary: a retrospective cohort study. CMAJ Open.

[9] Pheley A, Lois H and Strobl J (2006). Interests in research electives among osteopathic medical students. J Am Osteopath Assoc.

[10] Carter L, McClellan N, McFaul D, Massey B, Guenther E, Kisby G (2016). Assessment of research interests of first year osteopathic medical students. J Am Osteopath Assoc.

[11] Ramos K, Schager S, Tracz S (2003). Validation of the Fresno test of competence in evidence based medicine. BMJ.

[12] Smith A, Semler L, Rehman E (2016). A cross-sectional study of medical student knowledge of evidence-based medicine as measured by the Fresno test of evidence-based medicine. J Emerg Med.

[13] Cull W, Yudkowsky B, Schonfeld D, Berkowitz CD, Pan RJ (2003). Research exposure during pediatric residency: influence on career expectations. J Pediatr.

[14] Sambunjak D, Straus S, Marusic A (2006). Mentoring in academic medicine: a systematic review. JAMA.

[15] Committee on Pediatric Research (2014). Promoting education, mentorship and support for pediatric research. Pediatrics.

[16] Ashurst J, Galuska M (2016). Osteopathic physicians on editorial boards of major medical journals over the last 30 years. J Am Osteopath.

[17] Peppers B, Blumer J, Hostoffer R, Rowane MP (2018). National institutes of health and osteopathic medicine: another call for action and equality in a legal struggle won long ago. AAO Journal.

[18] Vinci R, Bauchner H, Finkelstein J, Newby PK, Muret-Wagstaff S, Lovejoy FH Jr (2009). Research during pediatric residency training: outcome of a senior resident block rotation. Pediatrics.

[19] Stevenson M, Smigielski E, Naifeh M, Abramson E, Todd C, Li S (2017). Increasing scholarly activity productivity during residency: a systematic review. Acad Med.

